# “I Want to Sleep, but I Can’t”: Adolescents’ Lived Experience of Sleeping Difficulties

**DOI:** 10.1177/1059840520966011

**Published:** 2020-10-16

**Authors:** Malin Jakobsson, Karin Sundin, Karin Högberg, Karin Josefsson

**Affiliations:** 1 Faculty of Caring Science, Work Life and Social Welfare, University of Borås, Sweden; 2 Department of Nursing, Umeå University, Sweden; 3 Department of Health Science, Karlstad University, Sweden

**Keywords:** adolescent, sleeping difficulties, lived experience, interview, phenomenological hermeneutic, school nurse

## Abstract

Sleeping difficulties are increasingly prevalent among adolescents and have negative consequences for their health, well-being, and education. The aim of this study was to illuminate the meanings of adolescents’ lived experiences of sleeping difficulties. The data were obtained from narrative interviews with 16 adolescents aged 14–15 in a Swedish city and were analyzed using the phenomenological hermeneutic method. The findings revealed four themes: feeling dejected when not falling asleep, experiencing the night as a struggle, searching for better sleep, and being affected the next day. The comprehensive understanding illuminates that being an adolescent with sleeping difficulties means it is challenging to go through the night and to cope the next day. It also means a feeling of being trapped by circumstances. As the adolescents’ lived experiences become apparent, the possibility for parents, school nurses, and other professional caregivers to support adolescents’ sleep increases.

Sleeping difficulties are recognized as an international public health issue (American Academy of Pediatrics, 2014; Chattu et al., 2018; Gradisar et al., 2011; Louzada, 2019). Research shows that 39–73% of adolescents get insufficient sleep night after night (Alves et al., 2020; Galland et al., 2020; Jakobsson et al., 2019; Twenge et al., 2017; Wheaton et al., 2018). The recommended 8–10 hr of sleep per night is essential for health, well-being, and education (Hirshkowitz et al., 2015). It is therefore highly relevant to illuminate adolescents’ lived experience of sleeping difficulties. The term sleeping difficulties has been used in a wide range of contexts and covers many problems related to poor sleep (Ghekiere et al., 2018). In this study, it is used to represent insufficient sleep, including trouble falling asleep, waking up at night, and sleep that leaves an individual unrested.

Adolescents’ sleep changes tremendously due to the homeostatic sleep–wake process and circadian changes that occur with delayed sleep onset and wake time, both of which emerge at puberty (Carskadon, 2011; Crowley et al., 2018). Adolescents also undergo psychosocial development characterized by existential thoughts, the search for sexual identity, various interests, relationships, and career choices (Erikson & Erikson, 2004), all of which may affect sleep habits. In addition, adolescents’ sleep is influenced by different contextual factors. Positive factors include physical activity, good sleep hygiene, parental limit setting, a good family environment (Bartel et al., 2015), and supportive school and friends (Maume, 2013). Negative factors include a negative family environment; pre-sleep worry; technology use (other than television); evening light; the use of caffeine, tobacco, and alcohol (Bartel et al., 2015); and demanded ties to school and friends (Maume, 2013). Further, when adolescents themselves articulate reasons that have a negative influence on their sleep they highlight stress, technology use, poor sleep habits, existential thoughts, unmet needs, and suffering from mental ill-health (Jakobsson et al., 2020).

The negative consequences of inadequate sleep have been revealed in several systematic reviews (Crowley et al., 2018; Shochat et al., 2014; Sun et al., 2019). These consequences include obesity; mood disturbance with the risk of depression and anxiety; behavioral problems, such as aggression or impulsivity; negative school performance, such as reduced learning ability, memory impairment, and hyperactivity; and increased risk-taking related to alcohol, drugs, and driving.

It is of utmost importance to advance research in adolescents’ sleep. Becker et al. (2015) noted that a range of studies with different methodological perspectives are needed to inform and understand adolescents’ sleep. To date, there are few studies in sleep research that take a qualitative approach. It is important to illuminate adolescents’ own experience of sleeping difficulties to acquire broader knowledge and the necessary evidence in order to provide preventive care interventions. The aim of this study is therefore to illuminate the meanings of adolescents’ lived experience of sleeping difficulties.

## Method

### Research Design

The study takes a phenomenological hermeneutic research approach. This approach is inspired by Ricœur’s (1976) interpretation theory, which was further developed as a research method by Lindseth and Norberg (2004). The aim here is to reveal the meanings of lived experience, the everyday experiences of individuals, through the interpretation of texts. The approach combines the phenomenological aims of describing and explaining lived experiences with the hermeneutical aims of understanding and interpreting the same. This method provides the opportunity to improve the understanding of adolescents’ lived experience of sleeping difficulties. The study was approved by the Swedish Ethical Review Board (Dnr: 2019-03806). Ethical research principles were followed carefully by fulfilling the requirements of information, consent, confidentiality, and usage (World Medical Association, 2013). If the participant is between 15 and 18 years of age and has the ability to understand what the research means for themselves, he or she can consent to be included in the research and the parents do not need to give consent, in accordance with The Swedish Code of Statutes (2003: 460, § 18). All adolescents gave written informed consent to participate in the study. Adolescents under 15 years of age also had their parent’s written informed consent to participate.

### Participants

The participants comprised *n* = 16 adolescents aged 14–15 with lived experience of sleeping difficulties. Sleeping difficulties consisted of insufficient sleep, trouble falling asleep, waking up at night, or sleep that leaves the adolescent unrested. None of the adolescents had sought specialist care or used sleeping pills for their sleeping difficulties. The participants have or have had experience of sleeping difficulties, with sleep lasting 3–7 hr per night, recurring since they started secondary school 2 years ago. The participants were in Grade 9, aged 14–15, which in Sweden is the last year of secondary school before applying for 3 years of upper secondary school. The 16 adolescents in this study comprised 10 boys and six girls (Table 1) from different demographic areas in a Swedish city. Twelve of the participants lived with cohabiting parents, 15 had siblings, and 12 participated in leisure activities. All but one stated they had friends with whom they spend time.

**Table 1.** Overview of Sample and Procedure.

Secondary School	I	II	III
Classes in Grade 9	4	4	7
Visited	1 Class, 22 adolescents	1 Class, 24 adolescents	3 Classes, 48 adolescents
Wanted to participate	1 Boy	7 Boys, 4 girls	8 Boys, 3 girls
Declined to participate	1 Boy	4 Boys	1 Boy, 1 girl
Participated	0	3 Boys, 4 girls	7 Boys, 2 girls

### Procedure and Data Collection

To collect data, the first author contacted the heads of school administration, who gave approval and informed the principals. The first author contacted three principals at three schools and informed them about the study; they also received the same information in written form. All the principals gave permission to conduct the study and provided the researchers with the names of teachers and school nurses. The teachers and school nurses were informed about the study via e-mail and suggested times for the first author to visit one class per school. However, at School III, three classes were amalgamated into one, as many students from each class were absent for tests (Table 1). The first author was present in the classroom, and the adolescents were informed about the study verbally and in writing; contact information was also given. The adolescents were then given a sheet of paper on which they wrote their name and mobile number and ticked a box if they wanted to participate in the study (*n* = 23), wanted to think and return (*n* = 9; none of these adolescents returned), or did not want to participate (*n* = 62). The first author collected the papers and told the adolescents she would contact those who indicated they wanted to participate to determine an interview day and time.

### Narrative Interviews

Sixteen audio-taped interviews were conducted in September to October 2019. Narrative interviews are especially useful in research concerning lived experiences (Lindseth & Norberg, 2004) and are therefore suitable for this study. The participants were asked to narrate their experiences of sleeping difficulties. The opening question was “Can you tell me about your sleep?” The adolescents were encouraged to narrate their lived experience of sleeping difficulties as freely as possible. Open-ended follow-up questions were used to clarify and encourage further narration, such as “Can you tell me more?” “How?” “When?” and “Can you give an example?” The interviews lasted 12–34 min (*m* = 24).

### Data Analysis

Using a phenomenological hermeneutic method (Lindseth & Norberg, 2004) means moving between the whole text and parts of the text through three interrelated phases: naive understanding, structural analysis, and comprehensive understanding. In the first phase, naive understanding, the transcribed interview text was read as a whole several times as open mindedly as possible. The aim was to interpret the initial understanding of the overall meanings of sleeping difficulties among adolescents. This phase was validated by the second phase, structural analysis. In this phase, the text was explained by dividing it into meaning units concerning adolescents’ experiences of sleeping difficulties. The meaning units were condensed and abstracted before searching for patterns that formed subthemes and themes (Table 2). In the third phase, comprehensive understanding, the subthemes and themes were summarized and critically reflected upon in relation to the aim and the context of the study. Further, the comprehensive understanding was a result of the combination of the naive understanding, the structural analysis findings, the authors’ preunderstanding, and insights from relevant literature and theories (Lindseth & Norberg, 2004).

**Table 2.** Example of the Structural Analysis.

Meaning Unit	Condensed Meaning Unit	Abstracted Meaning Unit	Subtheme	Theme
…when you have a hard time sleeping…when I have a hard time sleeping…really like this I want to sleep but I can’t, I have nothing to think about but I just can’t sleep and I sometimes wake up at night…when sleeping anxiously	I want to sleep but I can’t, I have nothing to think about but I can’t sleep	Feeling frustrated about not falling asleep	Being incapable of unwinding	Feeling dejected when not falling asleep

## Findings

The meanings of adolescents’ lived experiences of sleeping difficulties will be illuminated in three phases: naive understanding, structural analysis, and comprehensive understanding. The structural analysis includes participant quotes, with the participants being identified with a P and a number in parentheses.

### Naive Understanding

The interview text was read and reread as open-minded possible and with a phenomenological approach (Lindseth & Noberg, 2004) in order to obtain a sense of the material in its entirety and to initiate the approach for the structural analysis. This reading resulted in a naive grasp of the overall meaning of adolescents’ lived experiences sleeping difficulties.

The naive understanding of the meanings of adolescents’ lived experience of sleeping difficulties is being frustrated by difficulty falling asleep or by not getting enough sleep. Not falling asleep entails a mercilessly long wait for sleep without knowing how long that wait will be; this means a sense of despondency. The long wait means hours of loneliness in a quiet bedroom. It means being challenged to be in a quiet bedroom without distractions and having time to think. It means hours of fear and concerns about being misunderstood in communications with friends, parents, and teachers; failing in school; not being good enough as a friend, child, partner, or student; and making the wrong choices for the future. When adolescents become overly pensive at night, it generates a feeling of annoyance, annoyance that fear and concerns take over logical thinking. Thinking of the next day when not falling asleep means being stressed and frustrated. A night with less sleep means having trouble getting up the next morning, being tired and easily irritated, feeling sluggish in body and mind, and being unfocused in school the next day. When the brain is unable to relax despite being tired, it means feeling unpleasant. It is also unpleasant to let go of control, which is necessary to fall asleep. Searching for different ways to fall asleep faster means trying to keep control of sleep. When the attempt does not work, a sense of pointlessness occurs. When the attempt is successful, a feeling of hope occurs. Sometimes, the desire to be with friends, be on the internet, and be distracted before falling asleep is so great that not falling asleep and the next day’s consequences are ignored, which means a feeling of control.

### Structural Analysis

The structural analysis revealed four themes and nine subthemes (Table 3). The themes were feeling dejected when not falling asleep, experiencing the night as a struggle, searching for a better sleep, and being affected the next day.

**Table 3.** Overview of Themes and Subthemes.

Themes	Subthemes
Feeling dejected when not falling asleep	Being incapable of unwinding Feeling distressed when there are endless sleepless hours
Experiencing the night as a struggle	Feeling concerns growing when not falling asleep Being afraid of nightmares Wanting to experience belonging
Searching for better sleep	Evaluating various ways to fall asleep Seeking a sense of security
Being affected the next day	Feeling limited in daily life Becoming a worse self

#### Feeling dejected when not falling asleep

Feeling dejected when not falling asleep encompasses the subthemes *being incapable of unwinding* and *feeling distressed when there are endless sleepless hours.* This includes the adolescent’s knowledge that they need to sleep to be their best, yet they cannot fall asleep. A sense of dejection therefore emerges.

#### Being incapable of unwinding

Being incapable of unwinding means not having the ability to relax when it is time to sleep. Falling asleep immediately after doing homework, talking on the phone, watching something interesting on television, or being on the computer or on social media is considered difficult. The experience is that everything done before going to bed must be processed before it is possible to relax, something that can take hours. To do nothing but wait to fall asleep is tedious and uncomfortable. Just waiting feels like not taking advantage of time, and to be in a totally quiet bedroom feels odd. While waiting to fall asleep, looking at YouTube, Instagram, or TikTok or listening to relaxing sleep music may feel like the best distraction. Adolescents find it hard to turn off distractions and easy to become dependent on them; at the same time, distractions seem essential to unwind and fall asleep.

…and then maybe you pick up the phone again after trying to sleep for a quarter of an hour. Then it just becomes a vicious circle…(P16)

Difficulty unwinding can also include being afraid of letting go of control. Unwinding and falling asleep means no longer being able to control thoughts, surroundings, or updates on social media. The meaning of being incapable of unwinding is that the brain does not relax despite the adolescent trying to fall asleep. Waiting for the brain to unwind and to fall asleep can take several hours, night after night. This wait requires the adolescent to have patience and evokes feelings of frustration, dejection, and sadness. These feelings can provoke the concerns that something is wrong with them.

…usually it’s just that I’m not tired enough, or the body is, but I don’t know…the brain just doesn’t switch off or what to say…so…(P2)

#### Feeling distressed when there are endless sleepless hours

Feeling distressed when there are endless sleepless hours means a sense of fear of missing valuable hours of sleep and then being so tired the next day that the adolescent risk failing at school. Being tired and failing in school feels embarrassing and disastrous. The distress means feeling that the entire future rests on whether you pass an exam or get the grades needed to enter the right high school. Not being able to fall asleep means being stressed because the desire to be on top tomorrow is lost by not falling asleep. Furthermore, feeling distressed when there are endless sleepless hours means having doubts about the next day, whether less sleep will make it harder to perform at school and whether the adolescent will be able to meet teachers’ and parents’ requirements. To have others’ approval feels important and increases adolescents’ distress when sleeplessness continues.

…I get stressed, I feel like this—no! now I must fall asleep because I will be up soon. I have to sleep…I have an important test tomorrow so I can manage…(P1)

#### Experiencing the night as a struggle

Experiencing the night as a struggle encompasses the subthemes *feeling concerns growing when not falling asleep*, *being afraid of nightmares*, and *wanting to experience belonging.* These subthemes highlight feelings that adolescents with sleeping difficulties struggled with during the night and find hard to get rid of.

#### Feeling concerns growing when not falling asleep

Feeling concerns growing when not falling asleep means that thoughts and speculations prevail. Thoughts that are initially harmless grow into concerns and then unwanted ruminations. It means that adolescents brood and judge themselves and wonder whether they have done or said something wrong, if they could have behaved in some other way, or if they are good enough. Growing concerns raise the fear of not being able to manage the future, not having someone fall in love with them, not getting a job after finishing school, and not having a perfect life. When these existential thoughts grow into concerns, it means difficulties getting rid of them and falling asleep.

…I just keep thinking in several paths and it is like my thoughts get a lot bigger in the evenings than they are in the day…(P3)

Furthermore, when it is quiet and time to fall asleep, it is the first moment of the day the adolescents have the opportunity to think their own thoughts, which means that a whole day’s unthinkable thoughts pop up.

…I think it is a pretty big thing that so many of us young people have trouble sleeping, because we have no time to think and then the first time we shut down everything, in the outside world, that’s when we start thinking, kind of…(P7)

#### Being afraid of nightmares

Being afraid of nightmares means having such painful experiences of dreams that the adolescent dare not fall asleep. Dreams can include horrible situations such as kidnapping or rape. Having nightmares means waking up in a panic or even paralysis, a few minutes of fear when the body cannot move. When the dreams recur, the adolescent experiences fear and wonders whether the nightmares are a sign that something bad will happen. This fear makes it impossible to fall asleep.

…then I try to stay awake, because I do not want to fall asleep, I do not know how to explain but I do not want to fall asleep because I am afraid that I will have a nightmare again…(P 3)

#### Wanting to experience belonging

Wanting to experience belonging means staying awake during the night waiting for responses from friends on social media. Falling asleep during social interactions with friends means a perceived risk of being outside the social context, which is not an option. Having sleeping difficulties is perceived as normal among adolescents; this means it is experienced as acceptable not to sleep when others do not sleep. Doing and being like everyone else feels important in terms of the sense of belonging.

…the social requirement is that you…there are group chats and if we are writing something, then you have to join and participate…(P11)

#### Searching for better sleep

Searching for better sleep encompasses the subthemes *evaluating various ways to fall asleep* and *seeking a sense of security.* These subthemes highlight youthful attempts to get better sleep, sometimes successful and sometimes not.

#### Evaluating various ways to fall asleep

Evaluating various ways to fall asleep means that adolescents experience a desperate need to try different ways to sleep better because the sleeping difficulties are punishing. The various ways to sleep better came from random people or websites, friends, parents, the school nurse, or the adolescents themselves. It is easy to give up trying to fall asleep, as the results are not immediately apparent.

…I went to the school nurse and talked and she has printed papers with different things you can do, such as counting to 100 or counting seven’s table forward and then backwards until you fall asleep but that has not really helped…(P14)

Previous disagreeable experiences of not falling asleep means that the adolescent fears experiencing that again. Even when finally finding a way to fall asleep easier, adolescents have a feeling of insecurity and wonder whether the technique they found to help them fall asleep will continue to work over time.

#### Seeking a sense of security

Seeking a sense of security means seeking out people, places, and routines that are familiar and bring calm in order to sleep. It may be about sleeping next to one’s mother or father or a friend to enable a sense of security to be able to fall asleep at all.

…when I sleep with my mom I fall asleep in five seconds, I put my head on the pillow, she says good night and then I fall asleep. I have a lot easier to wake up with my mom too because I have simply slept better with her…(P1)

A sense of security also means having the same routines every night. Adolescents who have routines that parents maintain find that this provides balance and security, which leads to a good sleep. It could feel embarrassing with parents who are strict about sleep habits, but at the same time, it gives a sense of security.

#### Being affected the next day

To be affected the next day encompasses the subthemes *feeling limited in daily life* and *becoming a worse self.* These subthemes are about adolescents’ experience that the hours of sleep lost each night means limited school and social interactions.

#### Feeling limited in daily life

Feeling limited in daily life means that after a night of too little sleep, school performance suffers. The day after too little sleep begins with great difficulty waking up, followed by fatigue during the school day. This becomes particularly noticeable during lessons that require a great deal of thought. The brain’s capacity is impaired, and the usual motivation during lessons does not exist. This means that one’s thoughts float easily to something else, and a sense of pointlessness about being in school occurs.

…I am getting out of focus and I am getting sluggish…(P 10)

To cope with fatigue and concentrate on lessons is challenging. Things such as stamping the feet, jiggling the leg, or drumming a pen on the table might help but are perceived as disturbing by teachers and classmates. Feeling limited in life means experiencing less commitment and reduced energy in leisure time. Lost sleep at night means a loss of energy in terms of spending time with friends or participating in activities. Even the drive and desire to participate in usual workouts or athletic matches disappear.

#### Becoming a worse self

Becoming a worse self means that after too little sleep, adolescents have feelings of being disadvantaged and are often in a bad mood. Experiencing oneself as a worse self is an unpleasant feeling. The usually pleasant and happy person instead becomes quiet, more easily sad, angry, and irritated. The experience is that after a night with too little sleep, classmates or family members who giggle, ask something or comment on something, or just clear their throat provoke completely unwarranted feelings of annoyance. Being more sensitive feels unsafe, as the adolescents are afraid to get angry or annoyed for no real reason because they do not want to hurt anyone.

…I kind of get more sensitive. I get sad easier and angry easier…(P 13)

### Comprehensive Understanding and Discussion

Being an adolescent with sleeping difficulties means it is challenging to go through the night and then cope the next day. This can seem a never-ending challenge that comes with feelings of frustration, desperation, annoyance, distress, concern, despondency, dejection, sadness, and fear. Having sleeping difficulties means harboring a panorama of emotions during the night. Often, adolescents do not share their sleeping difficulties or the feelings they bring because they believe that sleeping difficulties are a normal part of adolescence. Experiencing sleeping difficulties and knowing that sleep is important makes it even more difficult to break the cycle of sleeplessness. It also means looking for ways out, such as ways to unwind. Although adolescents’ attempts may be small or unscientific, there is a longing for a good night’s sleep. Unfortunately, there are internal and external demands that get in the way, such as the demands of friends, school, social media, and societal norms.

The adolescents in this study often experienced difficulty unwinding. Trying to unwind and lie in a quiet bedroom is challenging and not using the time while trying to fall asleep evokes feelings of frustration. Adolescents’ experience of not being able to unwind, and especially their inability to put away their electronic devices, has been the topic of several previous studies (Godsell & White, 2019; Hedin et al., 2020; Quante et al., 2019) about sleep and sleep barriers. These studies have found that adolescents experience mobile phone and screen time as a barrier to getting a good night’s sleep. Scott et al. (2019) captured underlying drivers for bedtime social media use and found that adolescents experienced concerns over negative consequences for real-world relationships if they disconnect and fear social disapproval when violating norms around online availability. These findings are in agreement with some of the experiences of the adolescents in this study such as finding it difficult to unwind and let go of control vis-à-vis social media, experiencing concerns about saying or doing something wrong or not being good enough, and wanting to experience belonging. For the adolescents in this study, shutting down social media to avoid sleeping difficulties means a fear of not belonging the next day. This perspective means that adolescents can feel trapped between the need for sleep and the need to belong.

When the adolescents in this study feel their concerns growing, it means that initially harmless and usual thoughts grow into concerns and turn into unwanted rumination at night. It is known (Huang et al., 2020) that negative repetitive thinking is associated with difficulties falling asleep and depressed mood, and it seems more common in adolescents with a high level of perfectionism. The adolescents in this study did not explicitly express concern about being perfect, but the meanings in their narratives of the fear of not being good enough, the fear of letting go of control, the fear of not performing optimally in school, and the fear of not managing to live a perfect life is interpreted as perfectionism or wanting to cope with internal and external demands. The adolescents’ sleeping difficulties means spoken or unspoken demands, demands that feel incompatible with getting a good night’s sleep.

In different ways, adolescents’ experience that sleeping difficulties means demands and stress in relation to school performance. They experience stress and concern when sleeplessness continues and a fear of missing valuable hours of sleep the next day. In addition, adolescents know that they will not perform as well in school after too little sleep. Stress related to school and homework is a commonly mentioned reason for sleeping difficulties among adolescents (Gaarde et al., 2020, Jakobsson et al., 2020). This is the result of both their demands of themselves and external expectations (Jakobsson et al., 2020).

The results of this study indicate that being an adolescent with sleeping difficulties means a feeling of being trapped in internal and external demands that make it unable to unwind on one hand and long for a good sleep on the other. Being trapped does not mean the adolescents consider themselves victims, as they acknowledge they have many different circumstances to relate to and need to find a balance.

A deeper understanding of this study can be gained if considered through the lens of Bronfenbrenner’s (2005) bioecological theory. Bronfenbrenner’s bioecological theory indicates and describes that adolescents’ development occurs in the context of several interacting ecosystems that include their personal characteristics, and systems in the context they live and have relations in (microsystems, mesosystems, exosystems, macrosystems), and the time dimension (chronosystem). Adolescents’ sleep is impacted by these different bioecological systems (Short et al., 2019).

Bronfenbrenner’s bioecological theory is often illustrated with the individual at the midpoint surrounded by four systems; the first system is close to the individual and the last is not close to the individual but still affects them. Through all these systems is the fifth system, time (Bronfenbrenner, 2005). The adolescent with personal characteristics, such as age, gender, heredity, and biological condition, is the midpoint, surrounded by their closest relationships and activities, their microsystem, that is, parents, family, friends, school, and social media. The interrelationships between two or more microsystems comprise the second system, the mesosystem. The meaning of adolescents’ lived experiences of sleeping difficulties in this study can be understood based on how well their microsystem and their relationships within that microsystem function. For example, it is known that parental involvement in setting a bedtime and routines is essential for adolescents’ sleep (Bartel et al., 2015; Godsell & White, 2019). A lack of security, parental involvement, and routines may explain why the adolescents in this study find that their sleeping difficulties are related to *searching for better sleep* and security. Interactions with other microsystems, such as friends and social media, may give an understanding of adolescents’ experience in this study that sleeping difficulties mean *experiencing the night as a struggle*. The adolescents’ nightly struggle with concerns about how to fit in or experience belonging is perceived to be determined by how they interact with friends and social media and is thus dependent on the influence of these microsystems. School is another microsystem with which adolescents interact and thus informs some of the results in this study. It is known that school stress is associated with sleeping difficulties (Gaarde et al., 2020, Jakobsson et al., 2020); this can explain why the adolescents in this study who experience sleeping difficulties *affects the next day*. Being limited in school clashes with the requirement to get good grades, which in turn means stress and the inability of the adolescents to fall asleep. Outside the adolescents’ own context but nevertheless affecting them are the third system, the exosystem, which includes education system and political governance; the fourth system, the macrosystem, which includes norms, laws, and culture; and the fifth system, the chronosystem, which extends through all the other systems and make visible changes over historical time and development (Bronfenbrenner, 2005). The theme *feeling dejected when not falling asleep*, which is related to being incapable of unwinding, can be understood as being trapped in multifaceted circumstances. This includes a delayed sleep phase that emerges at puberty (individual); school start time (exosystem), which means that despite the fact that adolescents naturally will be tired later, they still need to go to school early; friends interacting on social media at night (microsystem); the norm to be available (macrosystem); and the historical time (chronosystem) that today’s adolescents grow up in, where everything goes on around the clock.

Based on the understanding that Bronfenbrenner’s bioecological theory offers, adolescents’ experiences of being trapped can be understood as a feeling of being in many different circumstances that may be difficult to relate to. Adolescents’ experience of sleeping difficulties in this study may thereby be explained by the preconditions and ability of adolescents to relate to and navigate in the contexts and circumstances around them.

The phenomenon of sleeping difficulties in adolescence is complex for both the adolescents who experience it and for those who research and treat it. A comprehensive approach is necessary to understand and support adolescents’ sleep. The bioecological theory (Bronfenbrenner, 2005) not only increases the understanding that adolescents’ experience of sleeping difficulties is far more complex than not falling asleep, but it also provides the insight that in order to support adolescents’ sleep parents, caregivers, and society need to genuinely listen to adolescents’ narratives about their own context. Listening to adolescents’ narratives about how they experience their sleeping difficulties is consistent with Ricœur’s (1992) theory on how narratives help us understand the motivation of human action and how people experience reality. By taking into account the systems that surround adolescents, including individual biological conditions and Bronfenbrenner’s systems, possible causes of adolescents’ sleeping difficulties can be made visible and measures put in place to resolve them.

## Methodological Consideration and Limitations

The trustworthiness of a qualitative study is achieved through credibility, dependability, confirmability, authenticity, and transferability (Guba & Lincoln, 1994). To ensure the credibility of this study, 16 adolescents, boys and girls from different demographic areas who have lived experiences of sleeping difficulties, were included. In phenomenological hermeneutic, 16 participants is a sufficiently large number, and the adolescents’ narratives were rich in their content, which enabled an in-depth interpretation. To increase the credibility, the analysis involved a critical and questioning approach by moving back and forth between the whole and the parts of the text. This movement shapes the three phases of the findings: naive understanding, structural analysis, and comprehensive understanding. Dependability was ensured by the method’s inherent validation. In order to achieve a truthful interpretation of the text, the process of interpretation was strict, and the naive understanding was validated by the structural analysis. The objectivity of the interpretation was considered during the process by the discussion of themes and subthemes between the authors and in research seminars and by reflecting the adolescents’ voices using quotes to ensure confirmability. The intention was to use everyday language to ensure that the text achieved authenticity, allowing readers to understand the findings. The sample was not chosen at random, which might be a weakness due to limited transferability. However, a clear method description facilitate the readers to evaluate the applicability of findings in other settings, context, or situations. The strength of the phenomenological hermeneutic method is the possibility to reveal the richness of the meanings of adolescents’ lived experience. The method does not aim to find a single truth (Lindseth & Norberg, 2004). This study presents possible meanings of a phenomenon and discloses truths about adolescents’ lived experience of sleeping difficulties.

## Implications for School Nursing

It is important to genuinely listen to adolescents’ experiences of sleeping difficulties, as their narratives include keys to prevent sleeping difficulties. These keys are in form of the persons experiences. Each adolescent has their unique experiences and by listening and asking questions in more depth, the school nurse and the adolescent together can figure out where sleeping difficulties arise and how to deal with them. Maybe the sleeping difficulties arise from being unable to unwind, a need of security, nightmares, concerns about school or friends, a missing of routines or other worries. Depending on where the sleeping difficulties are based, the support needs to be different. The adolescents’ narratives give school nurses the possibility to tailor unique and personal advice to the adolescents rather than giving general advice. When listening to the adolescent narratives, the school nurse empathy may increases, and this empathy may bring that the adolescents will be confirmed and thus help the adolescent to better handle their inability to sleep. School nurses and other school health professionals play a vital preventive role through nonmoralizing and attentive conversations with adolescents. School nurses also need to be aware of the adolescents’ context that they interact within and how it affects them. To have knowledge of the five systems in Bronfenbrenner’s bioecological theory enables a deeper understanding of circumstances that may affect adolescents’ sleep and thereby adds a further perspective to school nurses’ preventive work. Supporting conversations focusing on the adolescents’ experiences of sleeping difficulties, context, and circumstances might help the adolescents navigate and find a balance in relation to the circumstances that may affect their sleep. The findings in this study could help guide the assessment of sleeping difficulties. It highlights that as adolescents’ lived experiences become apparent, conditions may improve to support them in achieving better sleep. Additionally, our findings that the adolescents felt that their sleep is better if their parents continue to set boundaries and support them with routines indicate that involving parents is favorable. It would be of interest to continue with research into adolescents’ sleeping difficulties related to their context. Future research is suggested on how to support adolescents to navigate in their context, from the adolescents’ perspective.

## Conclusion

This study contributes new knowledge about the meaning of adolescents’ lived experience of sleeping difficulties. Being an adolescent with sleeping difficulties means it is challenging to get through the night and to cope the next day. In order to understand adolescents’ sleeping difficulties, a comprehensive understanding of the context in which the adolescents live is needed. Adolescents need to navigate and find balance in relation to circumstances that may affect their sleep and that are often beyond their control, such as norms and values in society, in social media, in school, and in family and friend groups. By genuinely listening to the adolescents’ narratives about their sleeping difficulties and the context in which they interact will parents, school nurses, other professional caregivers, and researchers increase their understanding. As the adolescents’ lived experiences become apparent, the conditions for supporting them achieve better sleep will improve.
